# Reviewed article: Costa AF, Tavares LF, Junior PCPC, Barbosa RBC, Honório OS, Oliveira ACR, Cardoso LO. Adaptation of a pediatric hospital in the city of Niterói, State of Rio de Janeiro, to the recommendations of the Brazilian Dietary Guidelines in 2023. Epidemiol Serv Saude. 2025:34;e20240532

**DOI:** 10.1590/S2237-96222025v34e20240532.a

**Published:** 2025-08-08

**Authors:** Marilia Mastrocolla de Almeida Cardoso

**Affiliations:** Hospital das Clinicas Botucatu

**Table:** 

Rating	Excellent	Good	Average	Below average	Poor
Interest			✓		
Quality			✓		
Originality			✓		
Overall			✓		

**Table:** 

Questionnaire	Yes	No	Not applicable
Does the manuscript contain new and significant information to justify publication?	✓		
Does the Abstract (Summary) clearly and accurately describe the content of the article?		✓	
Is the problem significant and concisely stated?		✓	
Are the methods described comprehensively?		✓	
Are the interpretations and conclusions justified by the results?		✓	
Is adequate reference made to other work in the field?	✓		

## Manuscript Structure

Length of article is: Adequate

Number of tables is: Too little

Number of figures is: Too little

Please state any conflict(s) of interest that you have in relation to the review of this paper (state “none” if this is not applicable): None

## Comments

O objetivo do estudo está claro e a temática é relevante para a área de alimentação no contexto hospitalar. No entanto, por questões metodológicas, seria interessante apresentar e detalhar de forma mais aprofundada exclusivamente a Fase 1.

Introdução : Seria interessante trazer na introdução dados sobre a questão no contexto da pediatria para reforçar a relevância do estudo.

Método: Está mencionada a autorização do CEP somente para a Fase 1, sendo que a Fase 2 necessitaria de aprovação do CEP pois envolveu a participação de pesquisadores, profissionais com expertise na área entre outros. Ainda sobre a Fase 2, seria muito importante descrever o processo de elaboração da recomendação (qual técnica foi utilizada durante o processo, como foi realizada a discussão com base nos dados coletados e o consenso para definir as recomendações). Especificamente para o Módulo 4 da Fase 1, os métodos de coleta e análise empregados precisam estar descritos com mais detalhes para que outros pesquisadores possam replicar e compreender como chegaram no resultado descrito. No módulo 2 da Fase 1 são citados escores para avaliação qualitativa da preparação - importante rever a citação que embasa esse escore. Os métodos para o cálculo dos resultados apresentados nos Quadros 1 e 2 não estão descritos com detalhes.

Conclusões: As conclusões sugerem que o resultado da avaliação do ambiente hospitalar foi satisfatório, porém não está claro quais foram os critérios para se chegar neste resultado final. 

